# The impact of the COVID-19 pandemic on the mood and family relationships of runners

**DOI:** 10.3389/fpsyg.2024.1295605

**Published:** 2024-04-05

**Authors:** Anna Vilaregut, Sixte Abadia, Sònia Pineda-Hernández, Sònia Torras, Xavier Pujadas

**Affiliations:** FPCEE Blanquerna, Ramon Llull University, Barcelona, Spain

**Keywords:** affect, runner, exercise dependence, family relations, COVID-19 lockdown

## Abstract

**Introduction:**

The aim of this study was to analyze the impact of the COVID-19 lockdown on the mood of amateur runners and on their relationships with their partners and families.

**Methods:**

Adult runners 18 years or older (*N* = 260) completed an online survey that included demographic information, standardized psychological assessments of Exercise Dependence (EDS-R) and mood (POMS), and, to measure relationship functioning, either the Dyadic Adjustment Scale (DAS) if they did not have children, or the Basic Family Evaluation Questionnaire (CERFB), measuring conjugal and parental relationships. Participants also answered questions about their exercise habits and the coping strategies they adopted during lockdown.

**Results:**

The results suggest that runners who saw the largest reductions in time spent exercising during lockdown tended to feel significantly less energetic (*p* < 0.05) and friendly (*p* < 0.01). In addition, they recorded significantly lower scores in marital satisfaction with their peers (*p* < 0.05). The runners with a higher degree of dependence on physical exercise registered significantly higher levels of depression, tension and anger than non-dependent runners (p < 0.001). Runners whose partners were physically active and did not have children had significantly higher scores marital satisfaction than runners whose partners were not physically active and had children (*p* < 0.05).

**Discussion:**

These findings seem to indicate that the psychological approach to athletes in the context of crises such as the pandemic should consider not only individual aspects, but also include the family perspective.

## Introduction

With more than 772 million confirmed cases worldwide (as of December 19, 2023, World Health Organization, 2023), COVID-19 is a global pandemic. When Spain reached 5,000 confirmed cases on March 14, 2020, the country’s government declared a State of Alarm (Real Decreto, 2020) and ordered the population to remain at home, except for those performing strictly essential duties.

Unlike most other countries around Europe, during this period Spain also prohibited outdoor exercise by individuals, and physical activity was limited to what could be done in the home (Rodríguez-Larrad et al., 2021). The State of Alarm continued through June 21, 2020, but the ban on outdoor physical activity was lifted on May 2, the start of a gradual easing of restrictions (Real Decreto-ley, 2020). During the lockdown, 41% of the country’s population did physical activity at least once a week, a drop of 13.8% from the figure for 2020 as a whole (Ministerio de Cultura y de Deporte, 2021).

Notwithstanding the epidemiological and medical benefits of the lockdown, a number of observers have pointed out the adverse effects these measures have had on the psychological well-being and behavior of the population. The months spent confined at home led to increases in confusion and stress (Alderwick et al., 2020), social isolation, loneliness, and the risk of self-harming behavior (Holmes et al., 2020), boredom, a lack of social contact, sleeping problems, anxiety disorders, depression, suicide, eating disorders, addictive behavior, domestic violence and child abuse (Bauer et al., 2020; Mengin et al., 2020). Balanzá-Martínez et al. (2021) studied lifestyle changes during lockdown and observed changes in environmental exposure (pattern of time spent indoors and outdoors), physical activity, stress management, social support, sleep patterns, diet and nutrition and substance abuse. These changes in habits and lifestyle prompted us to wonder how the lockdown affected people who view physical activity as an important part of their lives. More specifically, we were interested in whether the ban on outdoor physical activity that accompanied the State of Alarm declared on March 14, 2020, had affected the family and partner relationships of runners.

These changes in habits and lifestyle led us to ask how the lockdown affected people who view physical activity as an important part of their lives. And, if the ban on outdoor physical activity that accompanied the state of alarm declared on March 14, 2020 had affected the runners’ mood and family relations. These athletes were among the groups most directly affected by the restrictions imposed by the Spanish government, as during the lockdown they were unable to continue their usual exercise routines. The large impact on this group, and the fact that running is the second most frequent physical activity in the country (Subdirección General de Estadística y Estudios, 2020) were the main factors that drove us to focus the study on these athletes.

As Rodríguez et al. (2020) and Fennell et al. (2022) observed, exercise contributes to overall physical and psychological well-being, both of which are essential in overcoming the effects of COVID-19. Despite the potential benefits of exercise in the context of the pandemic, a systematic review by Stockwell et al. (2021) showed that most studies have recorded a decrease in physical activity during the lockdown period. Along the same lines, in an international study De la Vega et al. (2022) found that during the COVID19 pandemic habitual exercisers decreased their usual exercise volume to about half of the usual amount.

A number of researchers have explored the effects of the COVID-19 lockdown on the well-being of athletes. For example, Meyer et al. (2020) found that adults who stopped doing physical activity during the period of restrictions saw their mental health worsen. Lockdown was often particularly difficult for athletes because they were deprived of their usual opportunities for physical exercise. Therefore, they tended to miss the health benefits and the rest and recuperation that they habitually got out of their exercise routines. The blow was especially felt by dual career athletes, for whom the combination of physical activity and other tasks is important. This deprivation also affected athletes’ mental health (in the form of greater anxiety, depression, social dysfunction and loss of self-confidence), which in turn made it even more difficult for them to maintain their training regimens (Brand et al., 2020; Pillay et al., 2020). The lockdown also isolated athletes from teammates and colleagues, which also denied them access to the protective effect exerted by social interactions with teammates (i.e., social support and connectedness, Graupensperger et al., 2020). A lack of exercise has also often been linked to feelings of guilt and other negative emotions (Ackard et al., 2002; Malcolm and Velija, 2020; Cerea et al., 2023) and to less healthy eating habits (Taheri et al., 2023a). In light of the above, it is likely that athletes’ wellbeing was greatly affected by the pandemic.

Maltagliati et al. (2021) also observed a decrease in the frequency of physical activity in the context of COVID-19, especially among people who had strong exercise habits prior to the lockdown. Continuing to exercise in some form helped counteract some of the effects of the interruption of physical activity habits. Along similar lines, Petersen et al. (2021) recorded a decrease in physical activity among Australian adults during lockdown. The study also found a positive association between certain psychological constructs (social support, self-efficacy and autonomous motivation) and the tendency to physical activity during lockdown.

Another study by Washif et al. (2022) found that during the lockdown most athletes at different levels of competition and in a range of different sports had reduced the frequency, duration and intensity of their activities. These athletes tended to report feeling less motivated in this period because of the need to do remote training (to exercise alone, 53%) and the lack of competition (58%).

Elsewhere, however, Martin et al. (2021) observed an increase in the number of days of exercise done each week by people in the UK, and they found that the use of apps and online platforms for physical activity had become more widespread. Meanwhile, Rodríguez-Larrad et al. (2021) recorded a decrease in moderate to intense physical activity among students in Spain during lockdown but found that this decline was accompanied by a greater presence of “mind–body” exercises such as Pilates, Tai-Chi and Yoga. Nonetheless, the study found an overall increase in sedentary behavior and in the use of social media.

Iancheva et al. (2020) and Brand et al. (2020) explored how the interruption of people’s usual physical activity during lockdown affected their moods. Another study along similar lines by Ronkainen et al. (2021) looked at differences in mood among participants in different types of sport. This study found that when restrictions were lifted and outdoor activity became possible again, people who usually exercise in the gym or do extreme conditioning program training or Pilates were less likely to see an improvement in their moods than others whose usual activities were badminton, volleyball, hockey, running, orienteering and hiking. This might be because the former group were unable to return to their usual indoor activities in the early phases of the lifting of lockdown restrictions. Elsewhere, Taheri et al. (2023c) analyzed the impact of COVID-19 2 years after the start of the pandemic on the mental health and eating habits of elite and sub-elite athletes and found that elite athletes displayed better mental health profiles than their sub-elite counterparts, the former showing lower levels of depression, anxiety and stress.

Another study by Roberts and Lane (2021) looked at the lockdown’s effects on boxers, finding that it had taken a toll on athletes’ moods, as the participants reported increased feelings of anger, confusion, depression, fatigue and tension, as well as a reduction in vigor, mostly due to changes in their training regimens. Meanwhile, a study by Jaenes Sánchez et al. (2021) found that athletes who accepted the need for lockdowns and social isolation tended to experience more positive emotional states such as feelings of friendship. Meanwhile, athletes who lacked motivation during this period reported higher levels of stressful thoughts, more behavioral problems, and greater emotional upheaval (anger, fatigue, tension, and depression). The results of another study in a similar vein showed that sufficient levels of physical activity accompanied by a positive attitude with regard to the issues athletes had to face in the context of the COVID-19 lockdown acted as protective factors against sleeping problems (Taheri et al., 2023b). A number of studies have documented that athletes in individual sports differ from those in team sports in their self-regulation abilities (Jonker et al., 2010) and their adaptation strategies (Nicholls et al., 2007), as well as in certain individual traits (Correia and Rosado, 2019) and personality characteristics (Eagleton et al., 2007). The research by Taheri et al. (2023b) did not find any differences between amateur and elite athletes in terms of how likely they were to view the pandemic as stressful, challenging or threatening.

Aghababa et al. (2021) reported a significant positive correlation between exercise dependence during lockdown and positive moods, as well as a corresponding significant negative correlation between this dependence and worsened mood states. Meanwhile, the study found that compliance with lockdown measures and exercise dependence were the best predictors of the frequency and intensity of exercise during this period.

Recent years have witnessed an increase in research into exercise dependency among runners, an interest driven by the growing popularity of running. One of the instruments most frequently used to measure this phenomenon is the Exercise Dependence Scale (Hausenblas and Downs, 2002), the Spanish version of which was validated by Sicilia and González-Cutre (2011). Smith et al. (2010) used this tool to conclude that competitive runners were more likely than amateurs to show symptoms of exercise dependency. Researchers such as Lukács et al. (2019) and Zandonai et al. (2020) also employed this instrument and observed that increases and decreases in the frequency of physical exercise among amateur runners depend on the context of exercise and other variables such as loneliness and anxiety. Elsewhere, researchers such as Ruiz-Juan and Zarauz Sancho (2012) and Nogueira et al. (2021) using the Spanish version of the Running Addiction Scale (RAS-8) have found that runners who score higher for addiction tend to be those who register the longest distances run and most time spent on training.

Whitcomb-Khan et al. (2021) defined the moment of impasse due to COVID-19 as a “critical pause” in the lives of athletes, a time marked by feelings of loss (of physical conditioning, of routine, of motivation and of identity). However, the researchers observed that most of the athletes in the study, after an initial period of adaptation and acceptance, used the situation as an opportunity for growth. Indeed, researchers such as Martin et al. (2021) and Szczypińska et al. (2021) found that acceptance is the most common coping strategy adopted by athletes during the lockdown. Other strategies included having a sense of humor, active coping and self-distraction (Martin et al., 2021), as well as reliance on social support (Levine et al., 2022). Elsewhere, Kaur et al. (2020) observed that as athletes were gradually able to overcome the effects of the pandemic through adopting more constructive attitudes, they were also able to find alternatives to their usual physical exercise. Activities such as home workouts, yoga and meditation helped them to stay physically active and fit, and to improve their mental health. López-Bueno et al. (2020) found that the most common way for Spanish adults to exercise during the lockdown was in the home, observing that the use of social networks and apps on mobiles and other devices was also frequent. For example, some sought out alternatives such as virtual races or remote running clubs, which helped them to maintain their competitive motivation and offered opportunities for social support (DeJong et al., 2021).

In light of these findings, we wondered whether the act of discussing and sharing the effects of the deprivation of physical activity with athletes’ most immediate circle, such as family, might also have contributed to protecting their wellbeing in the context of lockdown.

With regard to this issue of COVID and family relationships (couple and parental relationships), Günther-Bel et al. (2020) studied the individual and family wellbeing of 409 people who were locked down with their partners and/or children during the first 3 weeks of restrictions imposed by the Spanish government. Nearly half of the individuals (49.2%) in the study had experienced a high degree of state anxiety, but a lesser presence of depression (4.6%) was recorded. In terms of family relations, the researchers found that greater anxiety and depression during lockdown were tied to decreased dyadic adjustment in couples and worsened parental functioning. In other words, the implications of lockdown went beyond the individual, as the situation also affected couple and family relationships.

Williamson (2020) carried out another study of couples during the early stages of the pandemic in March and April of 2020. The findings showed that the pandemic had exerted moderate effects on the participants’ ability to deal with conflict. Among couples with more positive functioning, satisfaction increased and maladaptive attributions decreased, while the opposite occurred among couples with lower functioning. Working along similar lines, Quezada et al. (2020) assessed the relationship between the marital satisfaction of 101 Mexicans who were living with their partners and their experience of the lockdown. The study showed that those who were more satisfied with their relationships were more likely to have felt calm and happy during the lockdown and less likely to have suffered blows to their happiness, health, physical condition or emotional well-being.

Gadermann et al. (2021) explored the impact of the early stages of lockdown on the mental health of families in Canada. The results showed that parents living with children under 18 were more likely to have experienced a deterioration of their mental health in this situation (44.3%) than the participants without children (35.6%).

Shum et al. (2023), conducted a qualitative study to examine the experiences of parents during the COVID-19 pandemic. The findings revealed the concerns and uncertainties that parents had to deal with, as well as feelings of mental exhaustion. Despite the challenges, some parents reported positive results experiences, such as strengthening family ties during the pandemic. In this line, Calvalho and Matias (2023) in their study on the impact of the COVID-19 pandemic and confinement on families, they also detected experiences of parental exhaustion of couples during confinement that contributed to decrease relationship satisfaction and increase conflicts.

At this point, we wondered about the impact on the mood of amateur runners with differing degrees of exercise dependence who were locked down for a month and a half due to the Spanish State of Alarm Decree of March 14, 2020, and if this deprivation had an impact on their couple and family relations. In order to answer the questions we have posed, we undertook this study with the following objectives: (a) to describe the effects of lockdown and the accompanying ban on outdoor exercise on the moods of amateur runners with differing degrees of dependency on physical exercise, (b) to explore how runners’ family relations were affected, (c) to examine the relationship between dependency on physical exercise, mood and family relations of amateur runners during the lockdown and (d) to discover what aspects of their usual physical activity they most missed and what coping strategies they adopted to make up for the loss of opportunities for physical activity during the lockdown.

## Method

### Participants

A total of 260 amateur runners from six different athletic clubs and associations in Spain were recruited to take part in this cross-sectional exploratory study with the help of members of the groups. The selection of participants took the form of intentional non-probability convenience sampling. In order to be included, potential participants had to: (a) be legal adults, (b) reside in Spain and (c) be habitual runners. The descriptive data of the participants are summarized in Table 1.

**Table 1:** Descriptive statistics.

Variables	*n* (%)
Male	178 (68.5)
Female	82 (31.5)
Age	
18–24 years	1 (0.4)
25–34 years	18 (6.9)
35–44 years	82 (31.5)
45–54 years	105 (40.4)
>55 years	54 (20.8)
Marital status	
Partners with children living together	133 (51.2)
Cohabitant partners without children	50 (19.2)
Divorced with children and have a new partner	14 (5.4)
Divorced with children and did not have a new partner	15 (5.8)
Lived alone	27 (10.4)
Lived with their parents	21 (8.1)
Partner physically active (living together or not)	
Active partner	152 (58.5)
Inactive Partner	82 (31.5)
Degree of dependence	
Non-dependent symptomatic	152 (58.5)
Non-dependent asymptomatic	92 (35.4)
Risk dependence	16 (6.1)

### Instruments

*Exercise Dependence Scale* [EDS-R, Downs et al., 2004; Spanish validation by Sicilia and González-Cutre (2011)]. Made up of 21 Likert-type items, where responses range from 1 (never) to 6 (always) and yields an overall dependency score (*α* = 0.92), a higher score indicates a greater risk of dependency. Give individual scores for each of the seven factors that make up the scale. The subscales tolerance (3-items, *α* = 0.73) is defined as either a need for increased amounts of exercise to achieve the desired effect (e.g., *I continually increase my exercise intensity to achieve the desired effect/benefits*), withdrawal (3-items, *α* = 0.85) the same amount of exercise is performed to relieve or avoid withdrawal symptoms (e.g., *I exercise to avoid feeling irritable*), intention effect (3-items, *α* = 0.83) represent when exercise is taken in larger amounts or over a longer period than was intended (e.g., *I exercise longer than intent*), lack of control (3-items, *α* = 0.78) is defined as a desire or unsuccessful effort to cut down exercise (e.g., *I am unable to reduce how long I exercise*), reductions in other activities (3-items, *α* = 0.68) assesses social, occupational, or recreational activities are given up or reduced because of exercise (e.g., *I would rather exercise than spend time with family/friends*), time (3-items, *α* = 0.84) represents a great deal of time is spent in activities necessary to obtain exercise (e.g., *I spend a lot of time exercising*) and continuance (3-items, α = 0.81) presents exercise that is continued despite knowledge of having a persistent or recurrent physical or psychological problem that is likely to have been caused or exacerbated by the exercise (e.g., *I exercise despite recurring physical problems*). It allows dividing athletes into three groups: risk of dependency, non-dependent-symptomatic, and asymptomatic non-dependent.

*Profile of Mood States* for adults (POMS, by McNair et al., 1971, 1992, Spanish validation by Andrade et al., 2013). This instrument assessment of an individual’s mood consists of 30 Likert-type items with responses ranging from 0 (Not at all) to 4 (extremely). Six different dimensions of mood are assessed over a given period of time. Four of these dimensions are negative: anger (5-items, *α* = 0.87, e.g., *angry*), fatigue (5-items, *α* = 0.87, e.g., *worn out*), tension (5-items, *α* = 0.88, e.g., *tense*) and depression (5-items, *α* = 0.86, e.g., *sad*). Two of them are positive: vigor (5-items, α = 0.86, e.g., *active*) and friendliness (5-items, α = 0.78, e.g., *friendly*).

*Basic Family Relations Evaluation Questionnaire* (*Cuestionario de Evaluación de las Relaciones Familiares Básicas,* CERFB; Ibáñez et al., 2012). This questionnaire consists of 25 items answered on a 5-point Likert scale with responses ranging from 1 (never) to 5 (always). It includes a 14-item conjugal functioning scale (*α* = 0.92), measuring the quality of parent–child relations (e.g., *I feel that my children return my affection*) and an 11-item parental functioning scale (α = 0.91), reflecting the quality of how parents relate to each other as a couple (e.g., *My partner knows how to treat me*). Scores for parental functioning can range from 1 to 55, while those for conjugal functioning can go from 1 to 70.

*Dyadic Adjustment Scale* (DAS; Spanier, 1976, Spanish adaptation by Martín-Lanas et al., 2017). This questionnaire consists of 32 items that measure people’s overall satisfaction with their relationships with their partners with whom they cohabitate, whether or not they are married. It consists of 4 subscales: consensus (13 items, *α* = 0.90) degree of agreement with the partner (e.g., *about career decisions*), cohesion (5 items, *α* = 0.86) degree of participation with the partner in activities together (e.g., *lough together*), satisfaction (10 items, *α* = 0.94) degree of satisfaction with the partner (e.g., *Do you confide in your mate?*) and affective expression (4 items, α *= 0.96*) *degree of agreement with the partner regarding emotional affections* (e.g.*, demonstration of affection*). The overall score for dyadic adjustment can range from 0 to 151. Higher scores indicate greater levels of dyadic adjustment between the members of a couple.

#### *Ad hoc* questionnaire

This instrument was created for the purposes of this study and gathered demographic data, information on participants’ job status and living situation, and data about athletic activity before and during lockdown. Data was also collected about whether or not the partner did physical exercise. Finally, included open-ended questions in which participants were asked what they most missed about their pre-lockdown exercise routines and what strategies they were using to compensate for the lack of opportunities to maintain their usual routines during the lockdown.

### Procedure

This project received the approval of the Research Ethics Committee of the School of Psychology, Education and Sports Sciences, Blanquerna, Ramon Llull University (certificate # 1920006P). Before completing the questionnaires, which were conducted online during the first period of lockdown, each participant received detailed information about the study’s purpose and procedure, including a guarantee of confidentiality, and each provided her or his informed consent.

The questionnaires were administered online using Google Forms. They were distributed with the help of contacts (mainly coaches) from the six participating athletic clubs and associations. The forms were completed totally anonymously. The overall questionnaire had a three-part structure. The first section consisted of demographic questions and items about participants’ job status, living situations, and athletic practices both before and during the lockdown. This first part concluded with the two open-ended questions from the *ad hoc* questionnaire. The second part of the form consisted of the EDS-R and the POMS (*N* = 260). The form concluded here for participants who lived alone, with their parents (*n* = 48) or divorced with children and did not have a new partner (*n* = 15).

The third and last section was answered only by the participants who indicated living as a partner, either without (*n* = 50), with children (*n* = 133) or divorced with children and have a new partner (*n* = 14). The three groups completed the DAS (*n* = 197), and those who had children together also completed the CERFB (partners with children, *n* = 133).

### Data analysis

Parametric tests were used to analyze the data from the sample, following the principle of the Central Limit Theorem (TCL). The Pearson correlation coefficient was calculated in order to analyze the relationships among the variables of the decrease in hours of exercise during lockdown, dependence on physical exercise, mood and family relations (conjugal and parental relationships). *T*-Student was used to compare the means recorded by runners with different exercise dependence profiles for hours of exercise, mood and family relations. Groups were compared with Bonferroni’s *p* values to identify if there were statistically significant differences between each of the groups. *T*-student was used to compare the conjugal and parental relationships of runners whose partners were physically active with those whose partners were not.

Structural equation modeling (SEM) was conducted through the maximum likelihood estimation method (ML). The following goodness-of-fit indices were obtained: goodness-of-fit index (GFI), and the normed fit index (NFI), and the root-mean square error of approximation (RMSEA). The cut of points for these indices were: equal to or higher than 0.90 for GFI and NFI and equal or lower than 0.08 for RMSEA (Steiger, 2007; Hu and Bentler, 2009). Version 26 of the statistics program SPSS for Mac and version JASP 0.15 were used to analyze the quantitative data.

The qualitative analysis conducted here focused on the responses to the two open-ended questions in the first section of the questionnaire, in which participants were asked what they most missed about their pre-lockdown exercise routines and what strategies they were using to compensate for the lack of opportunities to maintain their usual routines during the lockdown. Thematic analysis was used to identify repeated patterns of meaning within the textual data. The method used here consisted of the six-phase analytic procedure described by Braun and Clarke (2006): (1) familiarizing oneself with data, (2) generating initial codes, (3) searching for themes, (4) reviewing these themes, (5) naming and defining (6) reporting the results. An interrater reliability of *K* = 0.84 was calculated between the assessments of the researchers. Discrepancies were resolved through discussion to obtain full agreement. Separately, the frequency of the answers in each category was analyzed, reaching a consensus on the results of this process.

The choice to use mixed analysis also responded to the desire to achieve a broader understanding of the area of study through the use of both qualitative and quantitative results (Creswell and Clark, 2011). We used dichotomous variables that represented the presence (1) or absence (2) of certain qualitative categories in the participants’ answers in order to explore potential associations with the variables measured through the POMS, EDS-R, DAS and CERFB.

## Results

With regard to the study’s first objective, we observed that the participants scored higher than the population as a whole for tension, depression, anger and lower than the broader population for vigor and friendship (see Table 2).

**Table 2:** Descriptive data on mood (POMS).

	*n*	*M* sample	SD sample	*M* population*	SD population*
POMS tension	260	6.85	4.84	5.01	4.54
POMS depression	260	6.05	4.23	3.25	3.91
POMS anger	260	5.87	4.55	2.93	3.84
POMS vigor	260	8.89	4.33	10.48	4.31
POMS fatigue	260	5.09	3.94	5.45	4.48
POMS friendship	260	12.03	3.85	13.46	3.36

*Andrade et al. (2013).

Meanwhile, the runners displayed a statistically significant decrease (*d* = 0.77, *p* < 0.001) in the amount of time they spent exercising during the lockdown (*M* = 2.32, SD = 1.06) compared to before the pandemic (*M* = 3.18, SD = 0.84). Those who saw the greatest drop-off in the time spent running reported significantly lower levels of vigor and lesser levels of friendship (see Table 3). Those with the biggest declines in time spent on exercise also tended to have higher levels of tension, depression, anger and fatigue, although the results were not statistically significant in this regard.

**Table 3:** Pearson’s Correlation between decrease in hours of physical exercise during lockdown and mood (POMS).

	Decrease in hours of exercise during lockdown
POMS tension	0.107
POMS depression	0.066
POMS anger	0.099
POMS vigor	−0.254**
POMS fatigue	0.063
POMS friendship	−0.130*

**Results deemed statistically significant at *p* < 0.01; *p < 0.05.

Continuing with the first objective, the *t*-Student showed statistically significant differences (*p* < 0.001) in tension, depression and anger between non-dependent symptomatic runners and non-dependent asymptomatic, with an effect size of *d* = 0.55 for tension (*M* = 7.66, SD = 4.96 and *M* = 5.14, SD = 3.82), *d* = 0.51 for depression (*M* = 6.69, SD = 4.27 and *M =* 4.63, *SD =* 3.61) and *d* = 0.52 for anger (*M* = 6.58, SD = 4.77 and *M* = 4.33, SD = 3.56), respectively.

The study’s second objective as can be seen in Table 4 makes clear, runners with partners but without children recorded significantly higher scores for marital satisfaction than those with both partners and children. Additionally, runners with partners but without children registered significantly higher scores for dyadic adjustment than those that have been recorded for the Spanish population as a whole.

**Table 4:** Descriptive data on dyadic adjustment (DAS) and conjugal and parental relationships (CERFB).

	*M* sample without children	SD sample without children	*M* sample with children	SD sample with children	*p*	*d*	*M* population	SD population
DAS consensus	54.52	8.58	51.25	10.91	0.058	0.64		
DAS satisfaction	42.71	3.78	40.65	5.98	0.028	0.71		
DAS cohesion	18.34	4.27	18.14	4.15	0.076	0.37		
DAS affection	9.12	2.36	8.50	2.54	0.138	0.57		
DAS total	124.94	15.08	119.50	18.59	0.050	0.64	114.90*	17.50*
CERFB conjugal relationship			53.29	9.89			57.35**	8.98**
CERFB parental relationship			42.19	4.82			43.93**	5.90**

*Martín-Lanas et al. (2017) and **Ibáñez et al. (2012).

The runners who experienced the greatest decreases in the time spent running during the lockdown recorded significantly lower scores for marital satisfaction (DAS) and conjugal relationship (CERFB). Also, while the differences did not reach the threshold for statistical significance, runners with the largest drop-off in hours tended to score lower for consensus, cohesion and affection in their relationships with their partners (DAS) and to have more difficulties in their parental relationship (see Table 5).

**Table 5:** Pearson’s correlation decrease in hours spent running during lockdown and dyadic adjustment (DAS) and conjugal and parental relationship (CERFB).

	Decrease in hours of exercise during lockdown
DAS consensus	−0.073
DAS satisfaction	−0.213^**^
DAS cohesion	−0.011
DAS affection	−0.126
DAS total	−0.137
CERFB conjugal relationship	−0.185*
CERFB parental relationship	−0.039

**Results deemed statistically significant at *p* < 0.01; *p < 0.05.

It is worth noting that relationships were found for some of the individual dimensions of exercise dependence. Athletes who prior to the lockdown had done exercise to avoid bad moods, anxiety and tension (withdrawal), continued exercising despite having physical problems or injuries (continuance), experienced difficulties in reducing the length, frequency and intensity of exercise session (lack of control), and spent most of their free time exercising (time) recorded lower scores for affection toward their partners. Additionally, runners who prior to the pandemic had preferred exercising to spending time with their families or friends or to working (reduction in other activities) tended to register lower scores for conjugal harmony, consensus with their partners, expressions of affection and dyadic adjustment (see Table 6).

**Table 6:** Correlations between exercise dependence (EDS), mood (POMS), dyadic adjustment (DAS), and conjugal and parental relationships (CERFB).

	Conjugal relationship	Parental relationship	DAS consensus	DAS satisfaction	DAS cohesion	DAS affection	DAS total
EDS tolerance	0.022	0.041	0.049	0.123	0.059	−0.080	0.085
EDS withdrawal	−0.056	0.069	−0.048	0.058	−0.013	−0.157*	−0.039
EDS continuance	−0.083	−0.094	−0.090	0.015	−0.060	−0.182*	−0.107
EDS lack of control	−0.064	−0.016	−0.117	0.034	−0.079	−0.206**	−0.092
EDS reduction in other activities	−0.276**	−0.056	−0.204**	−0.073	−0.114	−0.202**	−0.194**
EDS time	−0.027	0.052	−0.098	0.104	−0.036	−0.159*	−0.061
EDS intention effect	−0.010	0.122	−0.186**	0.105	−0.033	−0.073	−0.093
POMS tension	−0.144	−0.084	−0.201**	−0.183*	−0.142*	−0.245**	−0.246**
POMS depression	−0.279**	−0.173*	−0.303**	−0.293**	−0.205**	−0.341**	−0.378**
POMS anger	−0.200*	−0.116	−0.217**	−0.227**	−0.150*	−0.259**	−0.294**
POMS vigor	0.340**	0.181*	0.142*	0.390**	0.262**	0.185**	0.315**
POMS fatigue	−0.147	−0.051	−0.143*	−0.161*	−0.106	−0.141*	−0.237**
POMS friendship	0.307**	0.339**	0.145*	0.371**	0.241**	0.153*	0.285**

**Results deemed statistically significant at *p* < 0.01; *p < 0.05.

The results also show a negative correlation between scores for dyadic adjustment (DAS TOTAL) and those for tension, depression, anger and fatigue during the lockdown. Scores for conjugal relationships negatively correlated with those for depression and anger. Meanwhile, dyadic adjustment and conjugal relationship showed positive correlations with the scores for vigor and friendship (see Table 6).

It is also worth highlighting that runners with children whose partners also did exercise tended to have more conjugal relationships with their partners than their peers whose partners did not exercise. Meanwhile, runners without children whose partners exercised displayed greater marital satisfaction, cohesion and dyadic adjustment than their counterparts whose partners were not physically active (see Table 7).

**Table 7:** Comparison between family relationships of runners whose partners are physically active and those whose partners are not.

	*M* (SD) Active partner	*M* (SD) Inactive partner	*t*	*p*	*d*
DAS consensus	53.00 (10.22)	50.59 (10.00)	1.49	0.139	0.26
DAS satisfaction	42.03 (4,75)	39.65 (6,33)	2.73	0.007**	0.46
DAS cohesion	18.90(3,80)	16.87 (4,88)	3.19	0.002**	0.58
DAS affection	8.80 (2,61)	8.45 (2,29)	0.90	0.369	0.18
DAS total	123.44 (16,31)	116.42 (19,28)	2.47	0.014*	0.45
Conjugal relationship	55.00 (9,12)	50.83 (10,52)	2.42	0.017*	0.46
Parental relationship	42.51 (4,82)	41.77 (4,82)	0.87	0.387	0.18

** Results deemed statistically significant at *p* < 0.01; *p < 0.05.

**Figure 1:**
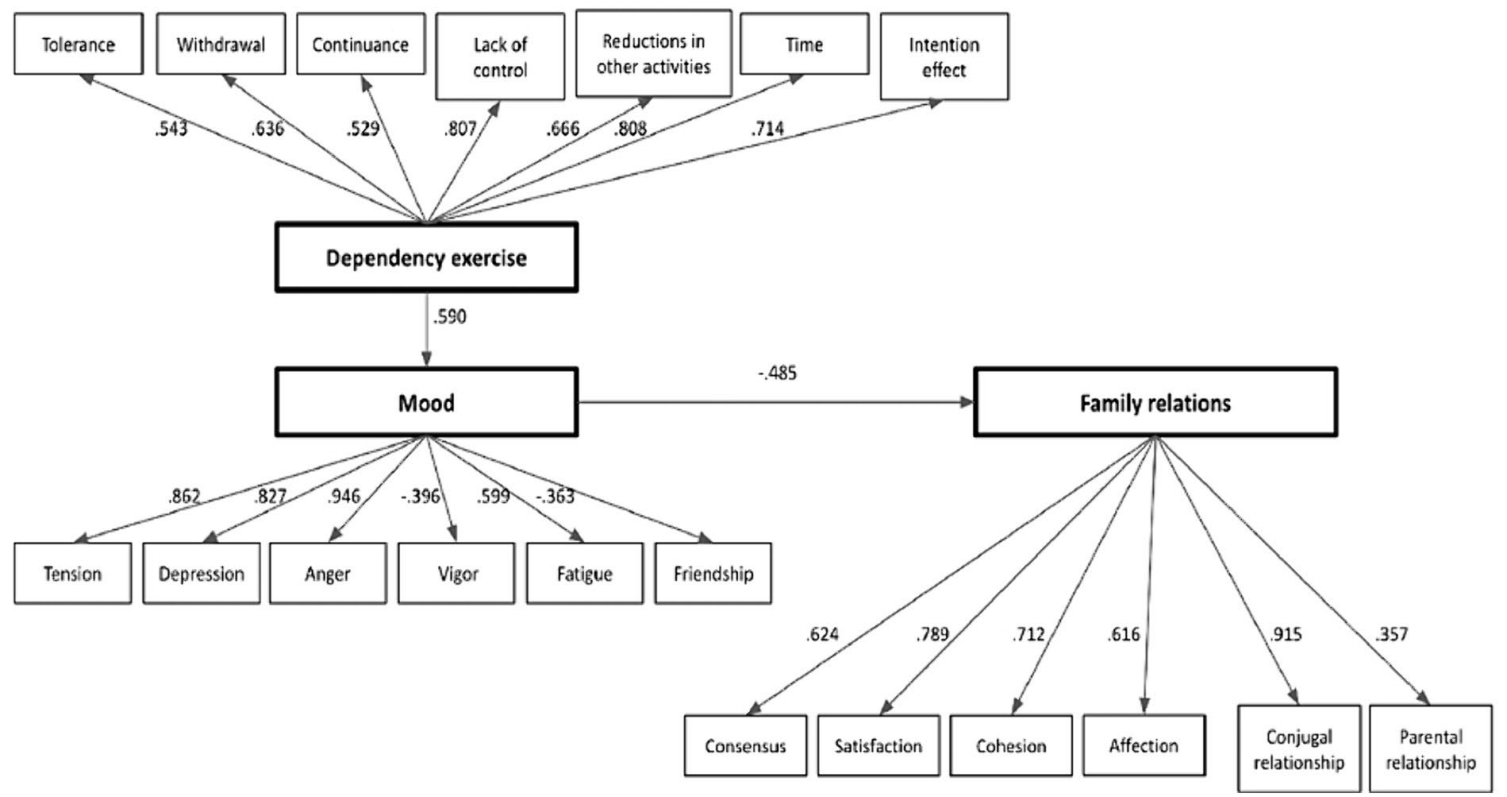
The structural equation model linking dependency on physical exercise with mood and family relations during the lockdown.

Regarding the third objective of this study, to examine the causal relationship between dependency on physical exercise with mood and family relations during the lockdown, a structural model was developed that included these constructs as latent variables. The proposed model included several indicators for each latent variable, and measurement errors and residuals were omitted from the path diagram. Goodness-of-fit indices revealed an adequate data fit: χ^2^ = 271.47, df = 117, GFI =0.99, CFI = 0.91, NFI = 0.85 and RMSEA = 0.07. Standardized regression weights were adequate in all cases, ranging from 0.35 to 0.94. According to this model, during the confinement, the runners’ dependence on physical exercise led them to have a state of mood with a high level of anger, depression and tension and less vigor and friendliness, which, in turn, affected their family relationships especially in the conjugal functioning, satisfaction marital and cohesion (see Figure 1).

Finally, regarding the last objective of this study, Table 8 shows some of the aspects that runners reported missing the most during the lockdown, grouped into categories with representative examples of each. The categories emerged through the qualitative analysis of the participants’ responses to an open-ended question about what they had most missed during the lockdown. The three overarching categories were nature/the outdoors, social relations and emotional benefits. This last category was divided into the three subcategories of relaxation, a feeling of freedom and self-esteem. The aspects runners most frequently reported missing during lockdown were the outdoors/nature and social relations.

**Table 8:** Aspects of previous physical activity that runners most missed during lockdown.

Category	Subcategory	Quote	Freq.
Outdoors/Nature		*Running in the mountains* *Being able to go out to run*	210
Social relations		*Being outdoors with the friends* *Socializing*	65
Emotional effects	Relaxation	*Exercising is stress relief* *Being able to relax mentally*	*8*
	Feeling of freedom	*Feeling free to go where I want* *Being able to feel free, which in turn makes me happy, something I cannot experience right now, unfortunately*	22
	Self-esteem	*Running helps me to think* *Winding up tired but proud of myself* *The psychological benefits that I get from the time I spend running*	22

Participants who identified social relations as the aspect they missed the most (*N* = 65) recorded significantly higher scores (*d* = 0.28, *p* = 0.049) for feelings of friendship (*M* = 12.77, SD = 3.22) during lockdown than those who did not report missing these relationships (*N* = 195; *M* = 11.77, SD = 4.04).

The comparison of the groups with different degrees of exercise dependence showed no statistically significant differences (*p* > 0.05) in the aspects of their pre-pandemic exercise routines that athletes most missed. The strategies that the participants reported using to compensate for the lack of opportunities for physical exercise during the lockdown are collected in Table 9.

**Table 9:** Strategies to compensate for the lack of physical activity during lockdown.

Category	Subcategory	Quote	**Freq.**
Running indoors		*Running in the corridor at home* *Running on the rooftop*	*14*
Alternative exercise	Personal resources	Going up and down the stairs I walk to work	149
	Virtual resources	Online training with my personal trainer and YouTube, videos from different platforms and virtual gyms	46
Wellness activities		Yoga, Chikung Pilates	24
Diet		A nutritious diet Regulating my diet	12
Strategies unrelated to exercise		Social networks and reading I devote most of my day to my daughter	15

It is worth noting that participants who ran indoors tended to score higher for tension (*d* = 0.51, *p* = 0.017; *M* = 8.86, SD = 6.53) and depression (*d* = 0.35, *p* = 0.016; *M* = 7.83, SD = 3.24) than those who did not (*N* = 246; M tension = 6.60, SD = 4.73; *M* depression = 5.82, SD = 3.51).

Meanwhile, participants who did alternative exercise during the lockdown scored significantly higher on the subscales measuring withdrawal (exercising to avoid bad moods, anxiety and tension) (*d* = 0.33, *p* = 0.011; *M* = 9.83, SD = 3.63) and time (spending most of their free time on exercise) (*d* = 0.35, *p* = 0.013; *M* = 9.16, SD = 3.68) than their counterparts who did not do any alternative exercise (*M* withdrawal = 8.57, SD = 4; *M* time = 7.92, SD = 3.44).

Runners who turned to wellness activities during the lockdown scored higher for conjugal relationship (*d* = 0.69, *p* = 0.030; *M* = 59.46, SD = 7.27), parental relationship (*d* = 0.62, *p* = 0.049; *M* = 44.91, SD = 5.99) and consensus with their partners (*d* = 0.57, *p* = 0.049; *M* = 56.82, SD = 6.34) than those who did not do these activities (*N* = 236; *M* conjugal relationship = 52.74, SD = 9.93; *M* parental relationship = 41.94, SD = 4.67; *M* consensus with partner = 51.94, SD = 10.34).

## Discussion

The lifestyle change brought about by the COVID-19 lockdown had a number of implications for psychological and physical health (Balanzá-Martínez et al., 2021). By way of further evidence of this, our study found that the interruption of runners’ habitual athletic activity affected their moods and their relations with their partners and families.

The low degree of prevalence of dependence on physical exercise among the participants in the study echoed the findings of Lukács et al. (2019), Zandonai et al. (2020), and Aghababa et al. (2021). Meanwhile, the runners in the study scored higher than the general population as a whole for tension, depression, anger, while they scored lower for vigor and friendship.

The findings with regard to tension coincide with those of studies by Jaenes Sánchez et al. (2021) and Roberts and Lane (2021). The level of tension also showed a correlation with the practice of running indoors as a strategy to compensate for the lack of physical activity during the lockdown. The runners in the study scored higher for depression than the population on average, another finding that confirms those of prior studies (Mengin et al., 2020; Pillay et al., 2020; Jaenes Sánchez et al., 2021; Petersen et al., 2021; Roberts and Lane, 2021). The presence of depression was statistically tied to the practice of running in enclosed spaces.

The decrease in the figures for friendship recalled the results of studies by Mengin et al. (2020), which found a reduction in social contact, Pillay et al. (2020), which observed a spike in social dysfunction, and Graupensperger et al. (2020), which detected a decrease in social relations among teammates. The importance of this decrease in contact with friends was reinforced by the fact that the runners in the study cited social relations as one of the pre-pandemic aspects of their athletic activities that they most missed. Indeed, Washif et al. (2022) made observations along the same lines, finding that solo training tended to reduce athletes’ motivation. Runners who did not adopt any athletic strategies in order to exercise during the lockdown were more likely to take up other activities that allowed them a greater feeling of friendly connection.

The comparison between groups of runners showed that the nondependent-symptomatic participants and the runners at risk of exercise dependence were more likely than nondependent-asymptomatic runners to suffer from tension and anger as in studies by Jaenes Sánchez et al. (2021) and Roberts and Lane (2021) and depression, echoing findings by Mengin et al. (2020), Pillay et al. (2020), and Petersen et al. (2021). These results stand in contrast with the general better mental health profile of elite athletes, who tend to have lower levels of depression, anxiety and stress than sub-elite athletes (Taheri et al., 2023c), often due to factors such as coping ability, income inequalities, and support.

These effects on mood coincide with the statistically significant decrease in the number of hours the participants spent on exercise in comparison with their pre-pandemic habits. These data align neatly with findings in studies by Aghababa et al. (2021), Maltagliati et al. (2021), Petersen et al. (2021), Rodríguez-Larrad et al. (2021), Stockwell et al. (2021), and Washif et al. (2022). The findings here stand in contrast, however, to those of a study by Martin et al. (2021). This drop in the number of hours spent on exercise corresponds with a decrease observed among the Spanish population in the wake of the ban on outdoor exercise by individuals (Ministerio de Cultura y de Deporte, 2021; Rodríguez-Larrad et al., 2021).

According to Iancheva et al. (2020), Jaenes Sánchez et al. (2021), and Ronkainen et al. (2021) this significant decrease in the number of hours the participants spent on exercise is connected to a decrease in motivation, as these groups of athletes were unable to keep up their usual exercise habits despite feeling the need to do so. This finding was reinforced by the fact that the most frequently cited factors that athletes said they missed during the lockdown were the outdoors/nature and social relations, as it seems reasonable to conclude that the lack of these aspects led to decreased motivation and, in turn, to a drop-off in hours spent on athletic activity.

However, the results also showed that the runners who spent the greatest amount of time on exercise both prior to the pandemic and during the lockdown were more likely to register higher degrees of exercise dependence, as was also observed by Aghababa et al. (2021) and in studies of runners conducted prior to the pandemic (Ruiz-Juan and Zarauz Sancho, 2012; Lukács et al., 2019; Zandonai et al., 2020; Nogueira et al., 2021). It is worth noting, however, that, much like Smith et al. (2010) found in their research, competitive runners showed greater symptoms of exercise dependence than non-competitive ones.

Meanwhile, athletes who had spent more of their time on running prior to the pandemic were likely to experience greater tension during the lockdown. As Iancheva et al. (2020) and Ronkainen et al. (2021) have written, this is due to the impossibility of continuing their usual exercise routine.

The findings described above connect neatly with the fact that the participants most often reported missing the outdoors/nature and social relations. These results also underline the importance of finding alternative ways of exercising to make up for the inability to continue usual regimens, beyond the acceptance of restrictive measures (Martin et al., 2021; Szczypińska et al., 2021; Taheri et al., 2023a,b). Runners who used this sort of coping strategy also tended to record better scores for withdrawal, tolerance and time (dimensions of dependence). It is also worth noting that a lack of exercise can contribute to an increase in negative mood states (Aghababa et al., 2021).

In Spain, unlike elsewhere in Europe, there was a complete ban on exercise outside the home. This undoubtedly led to a greater change in exercise habits, and it may have prompted athletes to adopt coping strategies. Some of the alternative activities that athletes did during this period included the use of the personal resources they had at hand by climbing up and down stairs or walking in the hallway. Others involved the use of digital resources such as devices and online platforms, a trend that has also been observed by others scholars (López-Bueno et al., 2020; DeJong et al., 2021; Martin et al., 2021) and is reflected in the data gathered by the *Encuesta de hábitos deportivos en España 202*0 [Survey of sporting habits in Spain 2020] (Ministerio de Cultura y de Deporte, 2021).

Meanwhile, athletes who spent the greatest amount of time exercising during the lockdown were more likely to feel energetic, cheerful, active and vigorous (vigor), and they displayed greater friendliness and understanding (friendship). It should be noted that athletes who participate in individual sports like running tend to have different kinds of self-regulation abilities (Jonker et al., 2010), coping strategies (Nicholls et al., 2007), individual characteristics (Correia and Rosado, 2019) and personality characteristics (Eagleton et al., 2007) than team sport athletes. The athletes in this study mostly turned to alternative physical activity (using personal and/or virtual resources).

Structural equation modeling confirmed the relationship between dependency on physical exercise, mood and family relations of amateur runners during the lockdown. According to this model, during the confinement, the runners’ dependence on physical exercise led them to have a state of mood with a high level of anger, depression and tension and less vigor and friendliness, which, in turn, affected their family relationships especially in the conjugal functioning, satisfaction marital and cohesion. The findings here, then, reaffirm the results of studies by Malcolm and Velija (2020) and Ackard et al. (2002), both of which tied decreases in physical activity to the presence of negative emotions.

As Günther-Bel et al. (2020) and Shum et al. (2023) observed, the effects of the lockdown were not only felt by individuals, but also affected couple and family relations. This was true among the runners in this study. The participants who felt angrier and more depressed registered lower scores for dyadic adjustment and conjugal functioning, while the athletes who reported more feelings of vigor and friendship had more harmonious relations in this regard.

Following Williamson (2020), we suggest that the lockdown’s impact on people’s satisfaction with their partners was lesser when their individual emotional state was better. In this study, we observed that runners who engaged in wellness activities tended to achieve more positive emotional states, as well as to display better consensus with their partners and more harmonious conjugal relations, and to maintain their parental functioning. It might also be the case, as the same author argues, that the pandemic saw improved satisfaction among couples with better functioning and decreased satisfaction among those functioning was less positive.

Our study also dovetails with findings by Aghababa et al. (2021) in that we found that athletes who spent more time on exercise during the lockdown were more likely to experience more positive mood states, a factor which, in turn, is linked to improved satisfaction with relationships. Our findings as to the differences between runners with and without children align with those of a study by Gadermann et al. (2021). Specifically, couples without children experienced greater satisfaction with their relationships and better dyadic adjustment during the lockdown than those with children. As Westrupp et al. (2021) point out, during the lockdown parents not only had to deal with work and other concerns, but also to take care of their children, and this often led to greater irritability and fewer positive expressions in family relations.

Finally, this study has made clear that runners whose partners also did physical exercise during the lockdown had better relations with these partners than runners whose partners were not physically active. As Linares and Campo (2002) have observed, sharing interests contributes to harmony among couples.

In short, our study has yielded exploratory results on the lockdown’s effects on the moods of a group of amateur runners, as well as on their relationships with their partners and families. In addition, it has been able to develop a structural model that examines the causal relationship between dependency on physical exercise with mood and family relations during the lockdown. It is important, of course, to be aware of the study’s limitations: (a) The responses to the online questionnaires were gathered from among amateur runners who all belonged to one of six athletic clubs and associations and, as such, were especially interested in the topic. This could limit the representativeness of the sample. (b) It should also be noted that a lot more men than women participated in the study, a trend that could have affected the results, (c) Although family relationships were examined, only one family member was interviewed which could result in a biased perspective, (d) Finally, it is worth highlighting that no in-depth interviews were conducted. Such interviews could have provided valuable information on some of the more subjective aspects of the study.

Despite these limitations, this study reveals the importance of analyzing the relationships of athletes and their families. Such an approach has the potential to contribute to knowledge about the effects of the pandemic and the accompanying lockdown on the world of sports. This study shows the way forward for future research in this area, which could explore how the impossibility of maintaining usual routines affected other groups of athletes in both individual and team sports. The study also points to the potential for other kinds of studies even outside the context of the pandemic, as there is relatively little research into the relations between participation in sports and athletes’ relationship and family dynamics. And finally, carry out longitudinal studies because moods can fluctuate over time and influence family relationships.

In conclusion, our study shows that the COVID-19 lockdown restrictions in Spain not only influenced amateur runners as individuals, but also influenced their relationships with their partners and families. The study shows that in situations of crisis like the pandemic, psychological interventions for athletes should bear in mind not only individual aspects, but also concerns about their most immediate relationships, such as those with their partners and families.

## Data availability statement

The raw data supporting the conclusions of this article will be made available by the authors, without undue reservation.

## Ethics statement

The studies involving humans were approved by Research Ethics Committee of the School of Psychology, Education and Sports Sciences, Blanquerna, Ramon Llull University (certificate # 1920006P). The studies were conducted in accordance with the local legislation and institutional requirements. The participants provided their written informed consent to participate in this study.

## Author contributions

AV: Writing – review & editing, Writing – original draft, Visualization, Validation, Supervision, Software, Resources, Project administration, Methodology, Investigation, Funding acquisition, Formal analysis, Data curation, Conceptualization. SA: Writing – original draft, Visualization, Supervision, Resources, Project administration, Methodology, Investigation, Funding acquisition, Conceptualization. SP: Writing – original draft, Supervision, Software, Methodology, Investigation, Conceptualization. ST: Writing – original draft, Validation, Software, Methodology, Investigation, Formal analysis, Data curation, Conceptualization. XP: Writing – review & editing, Writing – original draft, Visualization, Validation, Supervision, Resources, Project administration, Methodology, Investigation, Funding acquisition, Conceptualization.

## References

[ref1] Ackard D. Brehm B. Steffen J. (2002). Exercise and eating disorders in college-aged women: profiling excessive exercisers. Eat. Disord. 10, 31–47. doi: 10.1080/106402602753573540, PMID: 16864243

[ref2] Aghababa A. Badicu G. Fathirezaie Z. Rohani H. Nabilpour M. Zamani Sani S. H.. (2021). Different effects of the COVID-19 pandemic on exercise indexes and mood states based on sport types, exercise dependency and individual characteristics. Children 8:438. doi: 10.3390/children8060438, PMID: 34073668 PMC8225020

[ref3] Alderwick H. Dunn P. Dixon J. (2020). England’s health policy response to COVID-19. BMJ 369:m1937. doi: 10.1136/bmj.m1937 32414770

[ref4] Andrade E. Arce C. De Francisco C. Torrado J. Garrido J. (2013). Versión breve en español del cuestionario POMS para deportistas adultos y población general. Revist. Psicol. Deporte 22, 95–102.

[ref7] Balanzá-Martínez V. Kapczinski F. de Azevedo Cardoso T. Atienza-Carbonell B. Rosa A. R. Mota J. C.. (2021). The assessment of lifestyle changes during the COVID-19 pandemic using a multidimensional scale. Revist. Psiquiatría Salud Mental 14, 16–26. doi: 10.1016/j.rpsm.2020.07.003, PMID: 38620670 PMC7456305

[ref9] Bauer L. L. Seiffer B. Deinhart C. Atrott B. Sudeck G. Hautzinger M.. (2020). Associations of exercise and social support with mental health during quarantine and social-distancing measures during the COVID-19 pandemic: a cross-sectional survey in Germany. MedRxiv. doi: 10.1101/2020.07.01.20144105 PMC9013508 35472637

[ref10] Brand R. Timme S. Nosrat S. (2020). When pandemic hits: exercise frequency and subjective well-being during COVID-19 pandemic. Front. Psychol. 11:570567. doi: 10.3389/fpsyg.2020.570567, PMID: 33071902 PMC7541696

[ref9005] Braun V. Clarke V. (2006). Using thematic analysis in psychology. Qual. Res. Psychol. 3, 77–101. doi: 10.1191/1478088706qp063oa

[ref11] Calvalho M. Matias M. (Eds.) (2023). Parental exhaustion during COVID-19 pandemic: links to relationship outcomes and dyadic coping. Curr. Psychol., 1–11. doi: 10.1007/s12144-023-04658-2, PMID: 37359694 PMC10186299

[ref12] Cerea S. Pecunioso A. Casali N. Moro T. Paoli A. Ghisi M. (2023). How COVID-19 lockdown affected physical activity levels and wellbeing: an Italian survey. Int. J. Sport Exerc. Psychol. 21, 1054–1069. doi: 10.1080/1612197X.2022.2098358

[ref13] Correia M. E. Rosado A. (2019). Anxiety in athletes: gender and type of sport differences. Int. J. Psychol. Res. 12, 9–17. doi: 10.21500/20112084.3552 PMC7110169 32612783

[ref14] Creswell J. W. Clark V. L. P. (2011). Designing and conducting mixed methods research. 2nd Edn. Los Angeles: Sage Publications, Inc.

[ref15] De la Vega R. Almendros L. J. Barquín R. R. Boros S. Demetrovics Z. Szabo A. (2022). Exercise addiction during the COVID-19 pandemic: an international study confirming the need for considering passion and perfectionism. Int. J. Ment. Heal. Addict. 20, 1159–1170. doi: 10.1007/s11469-020-00433-7, PMID: 33293905 PMC7714253

[ref16] DeJong A. F. Fish P. N. Hertel J. (2021). Running behaviors, motivations, and injury risk during the COVID-19 pandemic: a survey of 1147 runners. PLoS One 16:e0246300. doi: 10.1371/journal.pone.0246300, PMID: 33577584 PMC7880469

[ref9004] Downs D. S. Hausenblas H. A. Nigg C. R. (2004). Factorial validity and psychometric examination of the exercise dependence scale-revised. Meas. Phys. Educ. Exerc. 8, 183–201. doi: 10.1207/s15327841mpee0804_1

[ref17] Eagleton J. R. McKelvie S. J. De Man A. (2007). Extra version and neuroticism in team sport participants, individual sport participants, and nonparticipants. Percept. Mot. Skills 105, 265–275. doi: 10.2466/pms.105.1.265-275 17918575

[ref18] Fennell C. Eremus T. Puyana M. G. Sañudo B. (2022). The importance of physical activity to augment mood during COVID-19 lockdown. Int. J. Environ. Res. Public Health 19:1270. doi: 10.3390/ijerph19031270, PMID: 35162293 PMC8835279

[ref19] Gadermann A. C. Thomson K. C. Richardson C. G. Gagne M. McAuliffe C. Hirani S.. (2021). Examining the impacts of the COVID-19 pandemic on family mental health in Canada: finding from a national cross-sectional study. BMJ Open 11:e042871. doi: 10.1136/bmjopen-2020-042871, PMID: 33436472 PMC7804831

[ref20] Graupensperger S. Benson A. J. Kilmer J. R. Evans M. B. (2020). Social (un)distancing: teammate interactions, athletic identity, and mental health of student-athletes during the COVID-19 pandemic. J. Adolesc. Health 67, 662–670. doi: 10.1016/j.jadohealth.2020.08.001, PMID: 32943294 PMC7489994

[ref21] Günther-Bel C. Vilaregut A. Carratala E. Torras-Garat S. Pérez-Testor C. (2020). A mixed-method study of individual, couple, and parental functioning during the state-regulated COVID-19 lockdown in Spain. Fam. Process 59, 1060–1079. doi: 10.1111/famp.12585 32678461 PMC7405150

[ref22] Hausenblas H. Downs D. (2002). How much is too much? The development and validation of the exercise dependence scale. Psychol. Health 17, 387–404. doi: 10.1080/0887044022000004894

[ref23] Holmes E. A. O’Connor R. C. Perry V. H. Tracey I. Wessely S. Arseneault L.. (2020). Multidisciplinary research priorities for the COVID-19 pandemic: a call for action for mental health science. Lancet Psychiatry 7, 547–560. doi: 10.1016/s2215-0366(20)30168-1, PMID: 32304649 PMC7159850

[ref24] Hu L. T. Bentler P. M. (2009). Cutoff criteria for fit indexes in covariance structure analysis: conventional criteria versus new alternatives. Struct. Eq. Model 6, 1–55. doi: 10.1080/10705519909540118

[ref25] Iancheva T. Rogaleva L. García-Mas A. Olmedilla A. (2020). Perfectionism, mood states, and coping strategies of sports students from Bulgaria and Russia during the pandemic COVID-19. J. Appl. Sports Sci. 1, 22–38. doi: 10.37393/JASS.2020.01.2

[ref26] Ibáñez N. Linares J. L. Vilaregut A. Virgili C. Campreciós M. (2012). Propiedades psicométricas del Cuestionario de Evaluación de las Relaciones Familiares Básicas (CERFB). Psicothema 24, 489–494. 22748745

[ref27] Jaenes Sánchez J. C. Alarcón Rubio D. Trujillo M. Peñaloza Gómez R. Mehrsafar A. H. Chirico A.. (2021). Emotional reactions and adaptation to COVID-19 lockdown (or confinement) by Spanish competitive athletes: some lesson for the future. Front. Psychol. 12:621606. doi: 10.3389/fpsyg.2021.621606, PMID: 34122217 PMC8187575

[ref28] Jonker L. Elferink-Gemser M. T. Visscher C. (2010). Differences in self-regulatory skills among talented athletes: the significance of competitive level and type of sport. J. Sports Sci. 28, 901–908. doi: 10.1080/02640411003797157, PMID: 20544490

[ref29] Kaur H. Singh T. Arya Y. K. Mittal S. (2020). Physical fitness and exercise during the COVID-19 pandemic: a qualitative enquiry. Front. Psychol. 11:590172. doi: 10.3389/fpsyg.2020.590172, PMID: 33250827 PMC7673425

[ref30] Levine O. Terry M. Tjong V. (2022). The collegiate athlete perspective on return to sport amidst the COVID-19 pandemic: a qualitative assessment of confidence, stress, and coping strategies. Int. J. Environ. Res. Public Health 19:6885. doi: 10.3390/ijerph19116885, PMID: 35682469 PMC9180442

[ref31] Linares J. L. Campo C. (2002). Sobrevivir a la pareja: problemas y soluciones. Barcelona: Planeta.

[ref32] López-Bueno R. Calatayud J. Andersen L. L. Balsalobre-Fernández C. Casaña J. Casajús J. A.. (2020). Immediate impact of the COVID-19 confinement on physical activity levels in Spanish adults. Sustain. For. 12:5708. doi: 10.3390/su12145708

[ref33] Lukács A. Sasvári P. Varga B. Mayer K. (2019). Exercise addiction and its related factors in amateur runners. J. Behav. Addict. 8, 343–349. doi: 10.1556/2006.8.2019.28 31146551 PMC7044555

[ref34] Malcolm D. Velija P. (2020). COVID-19, exercise and bodily self-control. Sociol. Deporte 1, 29–34. doi: 10.46661/socioldeporte.5011

[ref35] Maltagliati S. Rebar A. Fessler L. Forestier C. Sarrazin P. Chalabaev A.. (2021). Evolution of physical activity habits after a context change: the case of COVID-19 lockdown. Br. J. Health Psychol. 26, 1135–1154. doi: 10.1111/bjhp.12524, PMID: 33822454 PMC8250330

[ref36] Martin A. M. Champ F. Franklin Z. (2021). COVID-19: assessing the impact of lockdown on recreational athletes. Psychol. Sport Exerc. 56:101978. doi: 10.1016/j.psychsport.2021.101978, PMID: 34512179 PMC8422081

[ref37] Martín-Lanas R. Cano-Prous A. Beunza-Nuin M. I. (2017). DAS. Escala de Ajuste Diádico. Adaptación Española. Barcelona: TEA Ediciones.

[ref9001] McNair D. M. Lorr M. Droppleman L. F. (1971). Manual for the profile of mood states. San Diego, CA: Educational and Industrial Testing Service.

[ref9002] McNair D. M. Lorr M. Droppleman L. F. (1992). Revised manual for the profile of mood states. San Diego, CA: Educational and Industrial Testing Service.

[ref38] Mengin A. Allé M. C. Rolling J. Ligier F. Schroder C. Lalanne L.. (2020). Conséquences psychopathologiques du confinement. L'Encéphale 46, S43–S52. doi: 10.1016/j.encep.2020.04.007, PMID: 32370983 PMC7174176

[ref39] Meyer J. McDowell C. Lansing J. Brower C. Smith L. Tully M.. (2020). Changes in physical activity and sedentary behavior due to the COVID-19 outbreak and associations with mental health in 3.052 US adults. Int. J. Environ. Res. Public Health 17:6469. doi: 10.3390/ijerph17186469, PMID: 32899495 PMC7559240

[ref40] Ministerio de Cultura y de Deporte (2021). Encuesta de hábitos deportivos en España 2020. Secretaría General Técnica. Madrid: Ministerio de Cultura y Deporte.

[ref41] Nicholls A. R. Polman R. Levy A. R. Taylor J. Cobley S. (2007). Stressors, coping, and coping effectiveness: gender, type of sport, and skill differences. J. Sports Sci. 25, 1521–1530. doi: 10.1080/02640410701230479 17852669

[ref42] Nogueira A. Salguero A. Molinero O. Rosado A. Márquez A. (2021). Exercise addiction in competitive amateur runners. Int. J. Ment. Heal. Addict. 20, 2134–2150. doi: 10.1007/s11469-021-00504-3

[ref43] Petersen J. M. Kemps E. Lewis L. K. Prichard I. (2021). Promoting physical activity during the COVID-19 lockdown in Australia: the roles of psychological predictors and commercial physical activity apps. Psychol. Sport Exercise 56:102002. doi: 10.1016/j.psychsport.2021.102002, PMID: 36567740 PMC9760112

[ref44] Pillay L. Janse van Rensburg D. Jansen van Rensburg A. Ramagole D. A. Holtzhausen L. Dijkstra H. P.. (2020). Nowhere to hide: the significant impact of coronavirus disease 2019 (COVID-19) measures on elite and semi-elite south African athletes. J. Sci. Med. Sport 23, 670–679. doi: 10.1016/j.jsams.2020.05.016, PMID: 32448749 PMC7235602

[ref45] Quezada L. Landero R. González M. T. (2020). Couple satisfaction and impact of confinement by COVID-19 pandemic in Mexico. Interacciones 6:e173. doi: 10.24016/2020.v6n3.173

[ref5] Real Decreto (2020). Real Decreto 463/2020, de 14 de marzo, se declara el estado de alarma para la gestión de la situación de crisis sanitaria ocasionada por el COVID-19. Boletín Oficial del Estado, 67, de 14 de marzo de 2020, 25390 a 25400. Available at: https://www.boe.es/boe/dias/2020/03/14/pdfs/BOE-A-2020-3692.pdf

[ref6] Real Decreto-ley (2020). Real Decreto-ley 21/2020, de 9 de junio, medidas urgentes de prevención, contención y coordinación para hacer frente a la crisis sanitaria ocasionada por el COVID-19. Boletín Oficial del Estado, 163, de 10 de junio de 2020, 38723 a 38752. Available at: https://www.boe.es/boe/dias/2020/06/10/pdfs/BOE-A-2020-5895.pdf

[ref46] Roberts R. J. Lane A. M. (2021). Mood responses and regulation strategies used during COVID-19 among boxers and coaches. Front. Psychol. 12:624119. doi: 10.3389/fpsyg.2021.624119, PMID: 33746844 PMC7973043

[ref47] Rodríguez M. A. Crespo I. Olmedillas H. (2020). Exercising in times of COVID-19: what do experts recommend doing within four walls? Rev. Esp. Cardiol. 73, 527–529. doi: 10.1016/j.rec.2020.04.001, PMID: 32414660 PMC7142674

[ref48] Rodríguez-Larrad A. Mañas A. Labayen I. González-Gross M. Espin A. Aznar S.. (2021). Impact of COVID-19 confinement on physical activity and sedentary behaviour in Spanish university students: role of gender. Int. J. Environ. Res. Public Health 18:369. doi: 10.3390/ijerph18020369, PMID: 33418907 PMC7825050

[ref49] Ronkainen N. J. Pesola A. J. Tikkanen O. Brand R. (2021). Continuity and discontinuity of sport and exercise type during the COVID-19 pandemic. An exploratory study of effects on mood. Front. Psychol. 12:622876. doi: 10.3389/fpsyg.2021.622876, PMID: 33643151 PMC7907513

[ref50] Ruiz-Juan F. Zarauz Sancho A. (2012). Variables que hacen adicto negativamente a correr al maratoniano español (variables that makes negative addicted to run at Spanish marathoner). Retos 21, 38–42. doi: 10.47197/retos.v0i21.34602

[ref51] Shum A. Klampe M. L. Pearcey S. Cattel C. Burgess L. Lawrence P. J. Waite P. (2023). Parenting in a pandemic: a qualitative exploration of parents’ experiences of supporting their children during the COVID-19 pandemic. J. Fam. Stud., 29, 2335–2355. doi: 10.1080/13229400.2023.2168561

[ref52] Sicilia A. González-Cutre D. (2011). Dependence and physical exercise: Spanish validation of exercise dependence ScaleRevised (EDS-R). Span. J. Psychol. 14, 421–431. doi: 10.5209/rev_SJOP.2011.v14.n1.38, PMID: 21568198

[ref53] Smith D. Wright C. Winrow D. (2010). Exercise dependence and social physique anxiety in competitive and non-competitive runners. Int. J. Sport Exerc. Psychol. 8, 61–69. doi: 10.1080/1612197X.2010.9671934

[ref9003] Spanier G. B. (1976). Measuring dyadic adjustment: New scales for assessing the quality of marriage and similar dyads. J. Marriage Fam. 38, 15–28. doi: 10.2307/350547

[ref54] Steiger J. H. (2007). Understanding the limitations of global fit assessment in structural equation modeling. Personal. Individ. Differ. 42, 893–898. doi: 10.1016/j.paid.2006.09.017

[ref55] Stockwell S. Trott M. Tully M. Shin J. Barnett Y. Butler L.. (2021). Changes in physical activity and sedentary behaviors from before to during the COVID-19 pandemic lockdown: a systematic review. BMJ Open Sport Exerc. Med. 7:e000960. doi: 10.1136/bmjsem-2020-000960, PMID: 34192010 PMC7852071

[ref56] Subdirección General de Estadística y Estudios (2020). Anuario de estadísticas deportivas 2020. Madrid: Ministerio de Educación, Cultura y Deporte.

[ref57] Szczypińska M. Samełko A. Guszkowska M. (2021). Strategies for coping with stress in athletes during the COVID-19 pandemic and their predictors. Front. Psychol. 12:624949. doi: 10.3389/fpsyg.2021.624949, PMID: 33737896 PMC7960646

[ref58] Taheri M. Esmaeili A. Irandoust K. Mirmoezzi M. Souissi A. Laher I.. (2023a). Mental health, eating habits and physical activity levels of elite Iranian athletes during the COVID-19 pandemic. Sci. Sports 38, 527–533. doi: 10.1016/j.scispo.2023.01.002, PMID: 37362084 PMC10243596

[ref59] Taheri M. Irandoust K. Reynoso-Sánchez L. F. Muñoz-Helú H. Cruz-Morales K. N. Torres-Ramírez R.. (2023b). Effects of home confinement on physical activity, nutrition, and sleep quality during the COVID-19 outbreak in amateur and elite athletes. Front. Nutr. 10:1143340. doi: 10.3389/fnut.2023.1143340, PMID: 37139442 PMC10150803

[ref60] Taheri M. Saad H. B. Washif J. A. Reynoso-Sánchez L. F. Mirmoezzi M. Youzbashi L.. (2023c). Comparative study of the long-term impact of the COVID-19 pandemic on mental health and nutritional practices among international elite and sub-elite athletes: a sample of 1420 participants from 14 countries. Sports Med. 9:104. doi: 10.1186/s40798-023-00653-w, PMID: 37938473 PMC10632320

[ref61] Washif J. A. Farooq A. Krug I. Pyne D. B. Verhagen E. Taylor L.. (2022). Training during the COVID-19 lockdown: knowledge, beliefs, and practices of 12,526 athletes from 142 countries and six continents. Sports Med. 52, 933–948. doi: 10.1007/s40279-021-01573-z, PMID: 34687439 PMC8536915

[ref62] Westrupp E. M. Bennett C. Berkowitz T. Youssef G. J. Toumbourou J. W. Tucker R.. (2021). Child, parent, and family mental health and functioning in Australia during COVID-19: comparison to pre-pandemic data. Eur. Child Adolesc. Psychiatry 32, 317–330. doi: 10.1007/s00787-021-01861-z, PMID: 34417875 PMC8379590

[ref63] Whitcomb-Khan G. Wadsworth N. McGinty-Minister K. Bicker S. Swettenham L. Tod D. (2021). Critical pause: athletes’ stories of lockdown during COVID-19. Sport Psychol. 35, 43–54. doi: 10.1123/tsp.2020-0106

[ref64] Williamson H. C. (2020). Early effect of the COVID-19 pandemic on relationship satisfaction and attributions. Psychol. Sci. 31, 1479–1487. doi: 10.1177/0956797620972688, PMID: 33151125 PMC7797601

[ref65] World Health Organization (2023). Coronavirus disease (COVID-19) pandemic. Available at: https://www.who.int/emergencies/diseases/novel-coronavirus-2019 37068144

[ref66] Zandonai T. Manresa-Rocamora A. Monese L. Moya-Ramón M. Schena F. Chiamulera C. (2020). A descriptive study of exercise dependence: a short report among Italian and Japanese runners. J. Addict. Dis. 39, 133–137. doi: 10.1080/10550887.2020.1829450, PMID: 33028178

